# Feeding the Future: Next‐Generation Nutritional Frontiers—Innovations, Technologies, and Transformative Pathways for Sustainable Global Food Systems

**DOI:** 10.1002/fsn3.72157

**Published:** 2026-07-24

**Authors:** Zakari Adeiza David, Kareem Sarafadeen Olateju, Audu Godwin Amoka, Mustapha Omenesa Idris, Adefila Adebimpe Moyosore

**Affiliations:** ^1^ Department of Microbiology/Biochemistry Prince Abubakar Audu University Anyigba Kogi Nigeria; ^2^ Department of Microbiology College of Biosciences Federal University of Agriculture, Abeoukuta Ogun Nigeria; ^3^ Interdisciplinary Research Center for Membranes and Water Security King Fahd University of Petroleum and Minerals Dhahran Saudi Arabia; ^4^ Construction Department, Institute of Technical Training University of Applied Science Engineering and Technology Banjul The Gambia

**Keywords:** 3D food printing, alternative proteins, biofortification, cultivated meat, global food security, mycoprotein, novel foods, plant‐based proteins, precision fermentation, sustainable nutrition

## Abstract

The global food system faces compounding pressures: a world population projected to reach approximately 9.7 billion by 2050, an escalating burden of diet‐related non‐communicable diseases, persistent micronutrient deficiencies affecting approximately 2 billion people globally, and robust scientific evidence that conventional agriculture cannot sustainably expand within planetary boundaries. Against this backdrop, next‐generation food technologies have emerged as potentially transformative approaches to restructuring how humanity produces and consumes nutrition. This narrative review examines five technological pillars: (i) precision fermentation and microbially produced proteins, (ii) cultivated (cell‐based) meat and seafood, (iii) plant‐based, mycoprotein, and microalgae innovations, (iv) biofortification and functional food engineering, and (v) three‐dimensional (3D) food printing with novel ingredients. Each technology is evaluated for its nutritional profile, environmental footprint, scalability, regulatory status, and public health implications, alongside its documented limitations and scientific uncertainties. Cross‐cutting challenges, including regulatory fragmentation, consumer neophobia, energy intensity, equity of access, and citation integrity, are critically examined. The review concludes that while each pillar holds genuine promise, significant technical, regulatory, and socioeconomic uncertainties remain underemphasized in much of the existing literature. A synergistic, evidence‐anchored portfolio approach, grounded in sustainability science and guided by global health equity, is most credible. Priority research gaps are identified, including the need for long‐term dietary intervention trials, full‐system life cycle assessments incorporating renewable energy scenarios, and independent verification of bibliographic claims in rapidly evolving technology fields.

## Graphical abstract

Next‐generation food technologies—precision fermentation, cultivated (cell‐based) meat and seafood, plant‐based and mycoprotein innovations, biofortification and functional food engineering, and three‐dimensional (3D) food printing—are converging to address rising global food demand, persistent micronutrient deficiencies affecting approximately 2 billion people, and the environmental limits of conventional agriculture. This narrative review evaluates each technological pillar for its nutritional profile, environmental footprint, scalability, and regulatory status, alongside documented limitations and scientific uncertainties. A synergistic, evidence‐anchored portfolio approach—integrated with renewable energy systems and guided by global health equity—offers the most credible pathway toward safe, nutritious, affordable, and ecologically rational food at scale.

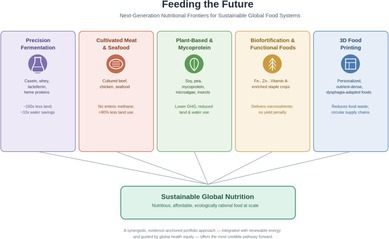

## 1 Introduction

The imperative to reconfigure food production is widely acknowledged across the natural and social sciences. Global food demand is projected to increase by 35%–56% by 2050, driven by population growth, rising incomes in low‐and‐middle‐income countries (LMICs), and dietary transitions toward animal‐source foods (van Dijk et al. 2021). Conventional agriculture currently occupies approximately 50% of habitable land and contributes an estimated 21%–37% of global greenhouse gas (GHG) emissions when the full supply chain is accounted for (Crippa et al. 2021); its continued expansion at historical rates would exert unsustainable pressure on freshwater systems, biodiversity, and the global climate (Willett et al. 2019).

Simultaneously, the global nutrition landscape reflects a well‐documented double burden. An estimated 2 billion people suffer from chronic micronutrient deficiencies in iron, zinc, selenium, and vitamin A, which impair cognitive development, immune function, and reproductive health and disproportionately affect populations in sub‐Saharan Africa and South Asia (Ramakrishnan 2002). Concurrently, diet‐related obesity, type 2 diabetes, and cardiovascular diseases are increasing in both high‐income countries (HICs) and, increasingly, in LMICs—driven by ultra‐processed food proliferation (Willett et al. 2019).

A constellation of next‐generation food technologies has emerged at the intersection of biotechnology, food science, materials engineering, and nutrition to address these twin challenges. These technologies represent foundational shifts: precision fermentation deploys engineered microorganisms as biofactories; cellular agriculture decouples meat production from animal slaughter; advanced biofortification delivers micronutrients directly through staple crops; and 3D food printing enables customized nutrition for vulnerable populations. Together, these approaches have been grouped in this review under the conceptual frame of “Next‐Generation Nutritional Frontiers,” a term chosen to acknowledge both their transformative potential and the genuine uncertainties that remain.

Several prior integrative reviews have addressed subsets of these technologies, including Kraak and Aschemann‐Witzel (2024) on plant‐based diets, Ofori et al. (2022) on biofortification, and Nielsen et al. (2024) on precision fermentation. The present review contributes an explicit multi‐technology synthesis that foregrounds critical analysis of limitations, cross‐cutting governance challenges, and a formal literature search methodology, elements that are absent or limited in prior comparable reviews. The aim is to inform researchers, policymakers, and food‐system practitioners of the current state of the science, key uncertainties, and pathways toward a food‐secure and ecologically viable future.

## 2 Literature Search Methodology

A systematic narrative review methodology was employed. Literature searches were conducted independently in PubMed, Scopus, Web of Science, and Google Scholar using primary search terms and Boolean combinations: “precision fermentation AND food proteins”; “cultivated meat AND life cycle assessment”; “cultivated meat AND scalability”; “plant‐based proteins AND sustainability”; “mycoprotein AND clinical trial”; “biofortification AND micronutrient deficiency AND LMICs”; “3D food printing AND personalized nutrition”; “alternative proteins AND regulatory”; and “food systems AND environmental impact AND greenhouse gas.” Additional targeted searches were performed for specific topics including “edible insects AND protein digestibility”, “nanoencapsulation AND bioavailability AND food”, and “precision fermentation AND market size”.

Inclusion criteria were: (i) peer‐reviewed original research articles, systematic reviews, or meta‐analyses; (ii) publication date between January 2012 and March 2026, with emphasis on 2019–2026 for technology and market data; (iii) English‐language publications; and (iv) direct relevance to at least one of the five technological pillars reviewed. Gray literature, including institutional reports (Menon and Olney 2024, GFI 2024, Department of Economic. (2024).) and industry reports, was selectively included for market data, regulatory developments, and program‐level statistics, and is identified as such in the text. Studies limited to laboratory‐scale proof‐of‐concept without clear relevance to scale‐up, human nutrition, or environmental sustainability were excluded. A total of approximately 150 sources were screened; 36 are formally cited in this article. All references were independently verified against primary sources prior to citation; no citations were included on the basis of secondary reporting or AI‐generated reference lists.

## 3 Conceptual Framework: The Five Pillars

**FIGURE 1:**
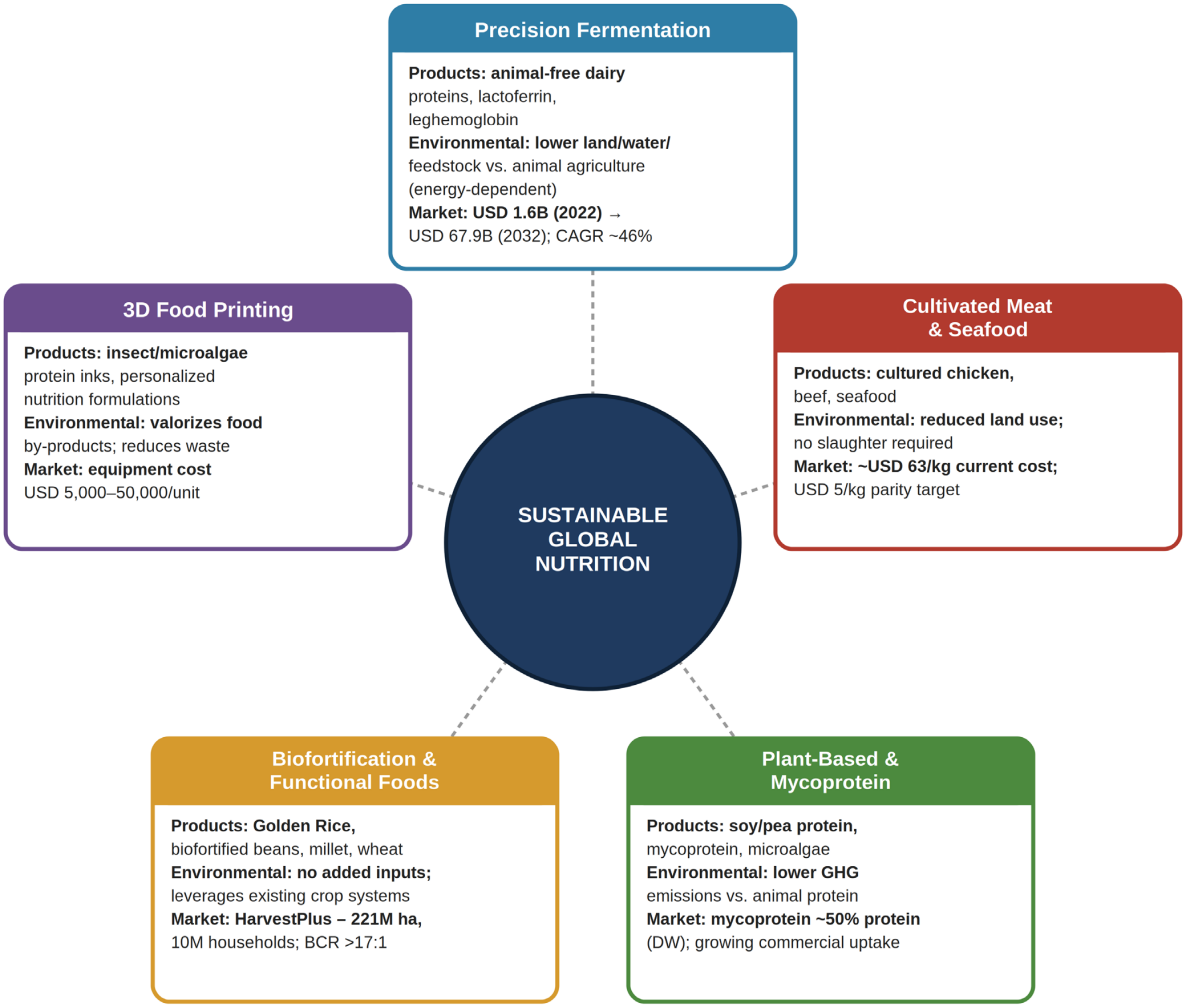
The five pillars of next‐generation nutritional frontiers. Each pillar (Precision Fermentation, Cultivated Meat and Seafood, Plant‐Based and Mycoprotein, Biofortification and Functional Foods, 3D Food Printing) converges toward the overarching goal of sustainable global nutrition. Key products, environmental advantages, market metrics, and supporting references are indicated for each pillar. CAGR, compound annual growth rate; DW, dry weight; LMICs, low‐and‐middle‐income countries.

The Next‐Generation Nutritional Frontiers framework rests on five interdependent technological pillars (Figure 1). Each pillar addresses distinct dimensions of the food‐nutrition‐sustainability nexus, yet all converge toward the overarching goal of producing safe, nutritious, affordable, and ecologically rational food at scale. Table 1 provides a comparative overview of these pillars with respect to key products, environmental advantages, and current market metrics with cited sources.

**TABLE 1:** Comparative overview of next‐generation food technologies.

Technology/Platform	Key products/Outputs	Environmental advantage	Market/Scale metrics
Precision fermentation	Casein, whey, lactoferrin, heme proteins, human milk oligosaccharides	Up to 100× more land‐efficient; ~10× water savings vs. animal farming	Market ~USD 1.6B (2022); projected ~USD 67.9B by 2032 at 46% CAGR (Knychala et al. 2024)
Cellular agriculture	Cultured beef, chicken, seafood	Eliminates enteric methane; reduces land use > 90%; reduces water use up to 78%	CM market ~USD 182 M (2022); projected CAGR 23.2%; cost currently ~USD 63/kg; target<USD 5/kg (Gu et al. 2025)
Plant‐based proteins	Soy, pea, mycoprotein, microalgae, edible insects	Lower GHG vs. conventional meat; reduced land and water use	Fastest‐growing alternative protein segment; driven by flexitarian demand (Kraak and Aschemann‐Witzel 2024)
Biofortification	Fe‐, Zn‐, vitamin A‐enriched staple crops	Delivers micronutrients within existing food supply; no yield penalty required	Cost‐effective; adopted on 221 M+ ha in LMICs; benefit: cost ratio > 17:1 (Ofori et al. 2022; Menon and Olney 2024)
3D food printing (3DFP)	Personalized nutrient‐dense foods; dysphagia‐adapted meals; insect/algae‐enriched snacks	Reduces food waste; valorizes by‐products; enables circular supply chains	Market projected USD 400 M+ by 2025; ~50% CAGR since 2018 (Zhang et al. 2024; Neamah and Tandio 2024)

*Note:* Summary of five core next‐generation food technologies including key products, environmental advantages, and market/scale metrics with direct source attributions.

Abbreviations: 3DFP, three‐dimensional food printing; CAGR, compound annual growth rate; CM, cultivated meat; LMICs, low‐and‐middle‐income countries; PF, precision fermentation.

## 4 Precision Fermentation: The Microbial Biofactory Revolution

### 4.1 Principles and Scope

Precision fermentation (PF) is the deployment of genetically engineered microorganisms, including yeasts, bacteria, and filamentous fungi, as biofactories for the targeted production of specific food‐relevant proteins, lipids, carbohydrates, vitamins, flavors, and other functional ingredients (Nielsen et al. 2024; Augustin et al. 2024; Eastham and Leman 2024). Unlike traditional fermentation, which relies on the natural metabolic repertoire of a microorganism, PF inserts heterologous genes encoding desired biomolecules into a microbial chassis, enabling production at concentrations many orders of magnitude above natural sources. The convergence of synthetic biology, genome sequencing, and advanced bioprocess engineering has progressively improved both the technical feasibility and commercial competitiveness of PF (Augustin et al. 2024).

Tubb and Seba (2019) projected that PF‐derived proteins could reach a cost target of approximately USD 10/kg by 2023–2025. Current analyses suggest this target remains aspirational for most molecule classes; recent techno‐economic modeling indicates production costs of USD 24–33/kg for some categories of PF molecules at current scale, underscoring the gap between projection and industrial reality (Eastham and Leman 2024). The global precision fermentation market was valued at approximately USD 1.6 billion in 2022 and is projected to reach approximately USD 67.9 billion by 2032 at a CAGR of approximately 46% (Knychala et al. 2024), though these projections carry significant uncertainty and depend on continued technical and regulatory progress.

### 4.2 Key Products and Nutritional Relevance

Among the most commercially advanced PF products are dairy proteins produced without cows. Animal‐free caseins and whey proteins, chemically identical to their bovine counterparts, can be expressed in engineered Komagataella phaffii (formerly Pichia pastoris) or *Bacillus subtilis* hosts and formulated into cheese analogs, yogurt, ice cream, and high‐protein beverages (Nielsen et al. 2024; Augustin et al. 2024). Lactoferrin, a bioactive iron‐binding glycoprotein with immunomodulatory and antimicrobial properties, represents another commercially targeted PF product with direct implications for infant formula and clinical nutrition (Nielsen et al. 2024). Beyond dairy, soy leghemoglobin produced by engineered yeast replicates the heme‐like flavor of beef in plant‐based burgers. Egg proteins, steviol glycosides for zero‐calorie sweetening, and human milk oligosaccharides for infant nutrition represent further active areas of PF development, each with distinct public health implications (Augustin et al. 2024; Eastham and Leman 2024).

### 4.3 Environmental Credentials and Uncertainties

PF‐based protein production systems are estimated to require substantially less land, water, and feedstock per unit of target protein compared with conventional animal agriculture (Tubb and Seba 2019). However, these estimates derive primarily from modeling studies using early‐stage production data rather than verified full‐scale industrial operations and should be interpreted with caution. A major unresolved uncertainty concerns energy consumption: PF at industrial scale requires substantial electricity for bioreactor operation, temperature control, aeration, downstream purification (chromatography, spray drying), and sterilization. Life cycle analysis of PF dairy proteins shows that when production is powered by the current average European electricity grid, GHG advantages over conventional dairy are present but substantially smaller than often claimed; when powered by renewable electricity, the advantage increases significantly (Augustin et al. 2024). Integration with decarbonized energy systems is therefore a prerequisite for, not an accessory to, realizing PF's environmental potential.

### 4.4 Challenges and Regulatory Landscape

Key technical challenges to PF scale‐up include achieving production titers above 50 g/L, a threshold several orders of magnitude above pharmaceutical protein manufacture standards, necessitating intensive strain engineering and bioprocess optimization (Nielsen et al. 2024). Downstream processing costs, including filtration, chromatographic purification, and spray drying, remain a major economic barrier to cost parity with conventional food ingredients (Eastham and Leman 2024). From a regulatory perspective, PF‐derived ingredients are categorized as “novel foods” under the European Food Safety Authority (EFSA) Novel Foods Regulation (EC 2015/2283), the US FDA Generally Recognized As Safe (GRAS) pathway, and Singapore's Food Safety Authority, each imposing different evidentiary requirements and timelines. The EFSA updated its novel food policy in early 2025, which is expected to accelerate approvals in Europe. Harmonization of these frameworks across jurisdictions is identified as a critical research and policy priority (Augustin et al. 2024).

## 5 Cultivated Meat and Seafood: Cellular Agriculture at the Plate

### 5.1 Scientific Foundations

**FIGURE 2:**
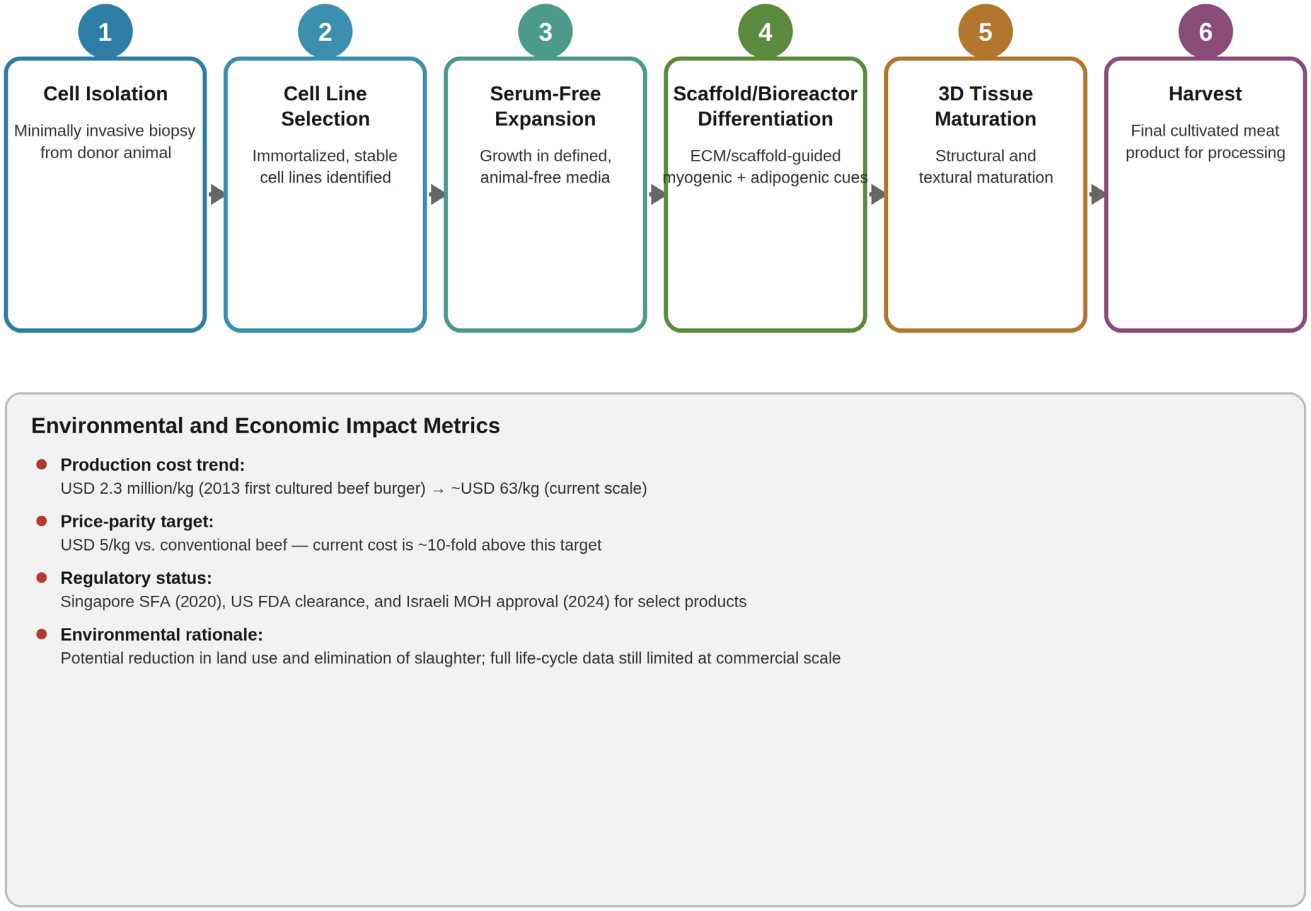
Cultivated meat production pipeline. Six sequential stages from cell isolation via minimally invasive biopsy through immortalized cell line selection, serum‐free expansion, scaffold/bioreactor‐guided differentiation, 3D tissue maturation, and harvest of the final product. Environmental and economic impact metrics are shown in the lower panel. ECM, extracellular matrix; CAGR, compound annual growth rate; TGF‐beta, transforming growth factor beta. *Sources:* Kim et al. (2025), Gu et al. (2025), Risner et al. (2025).

Cultivated meat (CM) is produced by isolating muscle progenitor cells from a donor animal via minimally invasive biopsy, expanding these cells in nutrient‐rich culture media, and differentiating them into muscle and adipose tissue within three‐dimensional scaffolds or bioreactors (Figure 2; Kim et al. 2025). The resulting product aims to replicate the cellular architecture, nutritional composition, and sensory attributes of conventional meat without requiring animal slaughter. Three interrelated regulatory phases govern this process: genetic regulation by myogenic transcription factors Pax7 and Myf5, and adipogenic factor PPARgamma; biochemical regulation via growth factors including IGF‐1, FGF‐2, and TGF‐beta; and mechanical cues from scaffold architecture and extracellular matrix stiffness that guide cellular alignment and maturation (Kim et al. 2025).

Regulatory milestones have been achieved in a small number of markets. Singapore's Singapore Food Agency was the first globally to approve cell‐cultured chicken for commercial sale in December 2020. Upside Foods and GOOD Meat received US FDA safety clearances for cell‐cultured chicken, and Aleph Farms received Israeli Ministry of Health approval for cultivated beef in January 2024 (Yun et al. 2024). Despite these approvals, commercial availability remains confined to extremely limited markets, and the technological and economic challenges of large‐scale production remain substantial. Production costs have fallen dramatically from the landmark USD 2.3 million per kilogram for the first 2013 cultured beef burger, and recent estimates place costs at approximately USD 63/kg at current scale, still roughly 10‐fold above the USD 5/kg price‐parity target with conventional beef (Gu et al. 2025).

### 5.2 Environmental and Public Health Dimensions

Life cycle assessments indicate that CM has the potential to reduce land use by over 90% and water use by up to 78% compared to conventional beef production, while eliminating direct methane emissions from enteric fermentation (Risner et al. 2025). However, these assessments carry critical caveats. CM production currently relies on highly refined, energy‐intensive culture media, and peer‐reviewed LCA data demonstrate that near‐term CM production powered by an average fossil fuel‐dependent electricity grid could exceed the GHG footprint of conventional beef in some scenarios, primarily due to the high energy intensity of maintaining sterile bioreactor conditions (Risner et al. 2025). The LCA advantages of CM therefore depend critically on the decarbonization of the electricity supply and the development of serum‐free, plant‐derived media at industrial scale, neither of which has been demonstrated commercially (Gu et al. 2025). From a public health perspective, CM theoretically reduces some zoonotic disease transmission risks and offers the opportunity to engineer healthier fatty acid profiles in the final product; however, both benefits require validation in standardized safety and nutritional studies that do not yet exist.

### 5.3 Scalability and Economic Barriers

The global cultivated meat market was valued at approximately USD 182 million in 2022 and is projected to grow at a CAGR of approximately 23.2% (Yun et al. 2024). Bridging the gap to conventional meat pricing requires reducing production costs to below USD 5/kg, a target dependent on simultaneous advances across multiple fronts: eliminating costly animal serum from culture media; developing immortalized cell lines with sustained myogenic potential and genomic stability; designing large‐volume (greater than 10,000 L) sterile bioreactors at acceptable capital cost; and scaling scaffold manufacture (Kim et al. 2025; Gu et al. 2025). AI‐assisted process optimization is being explored as a means to compress the development timeline for each of these fronts, though this remains at early‐stage demonstration. The withdrawal of regulatory approval for some CM products in certain US states illustrates that regulatory risk adds a further dimension of commercial uncertainty.

## 6 Plant‐Based Proteins, Mycoprotein, and Microalgae: The Green Protein Spectrum

### 6.1 The Rising Demand for Alternative Proteins

The growing adoption of flexitarian, vegetarian, and vegan dietary patterns, driven by environmental awareness, animal welfare concerns, and health motivations, has elevated plant‐based and fungal proteins within the alternative protein sector (Kraak and Aschemann‐Witzel 2024). Producing 1 kg of beef protein requires approximately 6 kg of plant protein input and generates, on average, approximately 20 times more GHGs than producing an equivalent mass of legume protein (Willett et al. 2019). However, growth trajectories for plant‐based meat markets vary substantially by region and market segment, and several major producers reported declining sales volumes in 2022–2024 following initial rapid growth, underscoring the difficulty of predicting consumer adoption trajectories. Yildiz and Bilgin (2024) identify persistent challenges, including off‐flavors, incomplete essential amino acid profiles, anti‐nutritional factors, and allergenicity, that must be systematically addressed for sustained mainstream adoption.

### 6.2 Soy, Pea, and Legume Proteins

Soy protein isolates (SPI) and concentrates remain the most commercially mature plant proteins, offering protein contents of 80%–90% on a dry weight basis and a complete essential amino acid profile. Anti‐nutritional factors (phytates, trypsin inhibitors, lectins), potential allergenicity, and persistent beany off‐flavors have stimulated research into alternatives (Yildiz and Bilgin 2024). Pea protein ( *Pisum sativum* ), typically 75%–85% protein on a dry weight basis, has gained considerable commercial traction due to its lower allergenicity, non‐GMO status, and compatibility with fermentation‐based flavor modification. A critical nutritional limitation of pea protein is its low methionine content, which may necessitate combination with complementary protein sources (e.g., rice protein) to ensure adequate provision of all essential amino acids (Yildiz and Bilgin 2024). Faba bean and chickpea proteins represent emerging alternatives with comparable nutritional profiles and growing agronomic interest. Chen and Carcea (2023) provide a comprehensive overview of advances in nutraceutical and functional plant‐based foods across these platforms.

### 6.3 Mycoprotein: Clinical Evidence and Limitations

Mycoprotein, produced by continuous aerobic fermentation of Fusarium venenatum in large stirred‐tank bioreactors and commercialized as Quorn, is nutritionally distinctive: approximately 50% protein on a dry weight basis with a complete essential amino acid profile, high dietary fiber content (predominantly beta‐glucans and chitin), and low saturated fat (Hashempour‐Baltork et al. 2020; Pavis et al. 2024). A randomized controlled trial by Pavis et al. (2024) involving 72 overweight, hypercholesterolemic adults demonstrated that a four‐week home‐based dietary intervention substituting meat with mycoprotein‐containing foods reduced serum LDL cholesterol by 10%, non‐HDL cholesterol by 6%, blood glucose by 13%, and c‐peptide concentrations by 27% compared to the meat control group. These findings extend previous laboratory‐controlled evidence into a real‐world setting and support mycoprotein as a clinically relevant dietary tool for cardiovascular and metabolic risk management. Limitations of the available evidence base include the short duration of trials, the involvement of industry funding in several studies, and the need for longer‐term dietary trials across more diverse populations.

### 6.4 Microalgae: Nutritional Potential and Production Constraints

Microalgae such as Arthrospira platensis (Spirulina) and *Chlorella vulgaris* represent a nutritionally dense protein source: protein content of 60%–70% dry weight, with omega‐3 fatty acids, B vitamins including B12, iron, and antioxidant carotenoids (Yuan et al. 2024). As photosynthetic organisms, microalgae fix atmospheric CO_2_ and can be cultivated in non‐arable environments using non‐potable water or even industrial effluents, conferring environmental advantages over land‐based crops. However, production at meaningful commercial scale remains capital‐intensive, requiring either open raceway ponds with higher contamination risk or closed photobioreactors with higher capital and energy costs. The characteristic taste, smell, and green color of microalgae present formulation challenges that substantially limit incorporation beyond supplement applications without extensive processing or flavor masking (Yuan et al. 2024). Their incorporation into 3D food printing inks is an active area of research that may help circumvent organoleptic barriers by embedding microalgae within flavored matrices. Table 2 provides a comparative nutritional profile of the key alternative protein sources.

**TABLE 2:** Comparative nutritional profiles of key alternative protein sources.

Protein source	Protein content	Key advantage	Primary limitation	Application area
Casein/Whey (PF)	85%–90% DW	Animal‐free; nutritionally identical to bovine counterparts	Allergenicity; high downstream processing cost	Dairy alternatives, infant formula
Soy protein isolate	80%–90% DW	Commercially scalable; low cost; complete AA profile	Beany off‐flavor; soy allergenicity; anti‐nutritional factors	Meat analogs, beverages, infant formula
Pea protein	75%–85% DW	Low allergenicity; non‐GMO; fermentation‐compatible	Incomplete AA profile (low methionine)	Beverages, snacks, meat analogs
Mycoprotein (Quorn)	~50% DW	Complete AA profile; high dietary fiber; clinically demonstrated LDL reduction (~10%) and blood glucose lowering	Consumer acceptance barrier; industry conflict of interest in some trials	Meat substitutes, cholesterol management
Microalgae (Spirulina/Chlorella)	60%–70% DW	Omega‐3 rich; vitamin B12; CO2 fixation; non‐arable cultivation	Taste/smell; green color; capital‐intensive production	Supplements, functional foods, 3DFP inks
Edible insects (Cricket/Mealworm)	55%–65% DW	Low land and water use; complete protein; locally sourced	Cultural neophobia; highly variable regulatory status by country	3D‐printed snacks, protein supplements

*Note:* Comparative profiles of six major alternative protein sources including protein content, key advantages, primary limitations, and principal application areas.

Abbreviations: 3DFP, three‐dimensional food printing; AA, amino acid; DW, dry weight; LDL, low‐density lipoprotein; PF, precision fermentation.

## 7 Biofortification and Functional Food Engineering: Closing the Micronutrient Gap

### 7.1 The Global Burden of Hidden Hunger

Chronic micronutrient deficiency, termed “hidden hunger,” is estimated to affect approximately 2 billion people worldwide, with iron and zinc deficiencies being the most prevalent globally (Ramakrishnan 2002). Iron deficiency anemia, the most common nutritional disorder worldwide, causes stunted physical and cognitive development, reduced work capacity, and elevated maternal and child mortality; zinc deficiency affects an estimated 17% of the global population and impairs immune function, growth, and wound healing (Hussain et al. 2022). Vitamin A deficiency remains a leading preventable cause of childhood blindness in LMICs. The UN Sustainable Development Goal 2 (SDG 2: Zero Hunger) explicitly targets elimination of all forms of malnutrition by 2030. Biofortification has been identified as one of the highest‐value food security investments, with benefit‐to‐cost ratios exceeding 17:1 for specific interventions in LMICs (Ofori et al. 2022).

### 7.2 Approaches to Biofortification and Evidence Base

Three biofortification strategies are currently deployed at varying scales. Agronomic biofortification applies zinc‐ or selenium‐enriched fertilizers to crops, or optimizes soil pH and microbial communities to improve micronutrient uptake. Conventional plant breeding selects and crosses cultivars with naturally elevated micronutrient concentrations. Transgenic and gene editing approaches, including CRISPR‐Cas9, introduce or upregulate specific biosynthetic or membrane transport genes to increase micronutrient content or bioavailability (Ofori et al. 2022; Kumar et al. 2022). Landmark achievements include Golden Rice (provitamin A), orange‐fleshed sweet potato (beta‐carotene), iron‐biofortified common beans and pearl millet, and zinc‐biofortified wheat (Ofori et al. 2022). The HarvestPlus program, operated by CGIAR, has deployed biofortified crop varieties on over 221 million hectares across LMICs, reaching an estimated 10 million farming households (Menon and Olney 2024).

The evidence base from human trials is notable for its strength relative to the other pillars reviewed. Randomized controlled trials across sub‐Saharan Africa and South Asia have demonstrated statistically significant improvements in serum iron, ferritin, and hemoglobin concentrations following consumption of iron‐biofortified pearl millet and beans in children and women of reproductive age, translating into measurable reductions in anemia prevalence and improvements in child growth outcomes (Ofori et al. 2022). However, critical limitations warrant acknowledgment. Micronutrient bioavailability from biofortified crops depends substantially on the food matrix, cooking method, and the presence of anti‐nutritional factors (phytate, polyphenols, oxalate) that may chelate iron and zinc; these interactions are incompletely characterized in real‐world dietary contexts (Ofori et al. 2022; Tang et al. 2009). Consumer acceptance of biofortified crops with altered sensory properties requires active promotion strategies. Regulatory frameworks for gene‐edited crops diverge substantially, with the European Court of Justice's 2018 ruling classifying CRISPR‐edited plants under the full GMO Directive, while the US treats many gene‐edited varieties similarly to conventional breeding, creating significant market access barriers for biofortified crops developed through gene editing (Puchta 2023).

### 7.3 Functional Foods and Nanoencapsulation

Beyond crop‐level biofortification, the industrial production of functional foods, defined as foods that provide clinically substantiated health benefits beyond basic nutrition through the incorporation of bioactive compounds, represents a parallel and complementary approach (Yuan et al. 2024). Nanoencapsulation has emerged as a key enabling technology, entrapping hydrophobic or chemically labile bioactive compounds within lipid nanoparticles, protein‐based nanocarriers, or carbohydrate nanostructures that protect them from oxidation, pH‐induced degradation, and enzymatic breakdown during gastrointestinal transit, while enabling controlled release at target sites (Yuan et al. 2024; Tan and Abd El‐Aty 2024). Specific applications include nanoencapsulated omega‐3 fatty acids resistant to oxidative rancidity, curcumin with enhanced bioavailability via lipid nanoparticles, and pH‐sensitive probiotic delivery systems that release bacteria selectively in the colon (Tan and Abd El‐Aty 2024). Cold plasma treatment and edible coating technologies further extend the shelf life and surface bioavailability of bioactive ingredients in fresh produce and minimally processed foods. Nonetheless, the regulatory status of nanoencapsulated food ingredients is unresolved in many jurisdictions, long‐term safety data at relevant human exposure levels remain limited, and the cost of nanoencapsulation technologies currently restricts their application largely to premium and clinical nutrition products.

## 8 Three‐Dimensional Food Printing: Precision Nutrition by Design

### 8.1 Principles and Technology Landscape

Three‐dimensional (3D) food printing (3DFP), also termed additive food manufacturing, is the layer‐by‐layer deposition of food materials guided by digital design files to create customized food structures with controlled geometry, texture, nutritional composition, and visual appearance (Neamah and Tandio 2024). The principal printing modalities are extrusion‐based printing (most widely adopted; suitable for pastes, gels, and doughs at viscosity ranges of 10–1000 mPa/s), inkjet printing (suitable for low‐viscosity materials at 1–30 mPa/s; resolution 20–200 μm), and selective laser sintering (SLS; suitable for powder‐based materials with high structural complexity achievable) (Neamah and Tandio 2024; Zhang et al. 2024). Each modality imposes distinct rheological requirements on the print ink. The global 3D food printing market was projected to exceed USD 400 million by 2025, with an approximate CAGR of 50% since 2018 (Zhang et al. 2024; Neamah and Tandio 2024), though these projections should be treated as indicative given the early stage of commercial adoption.

### 8.2 Novel Protein Inks: Insects and Microalgae

**FIGURE 3:**
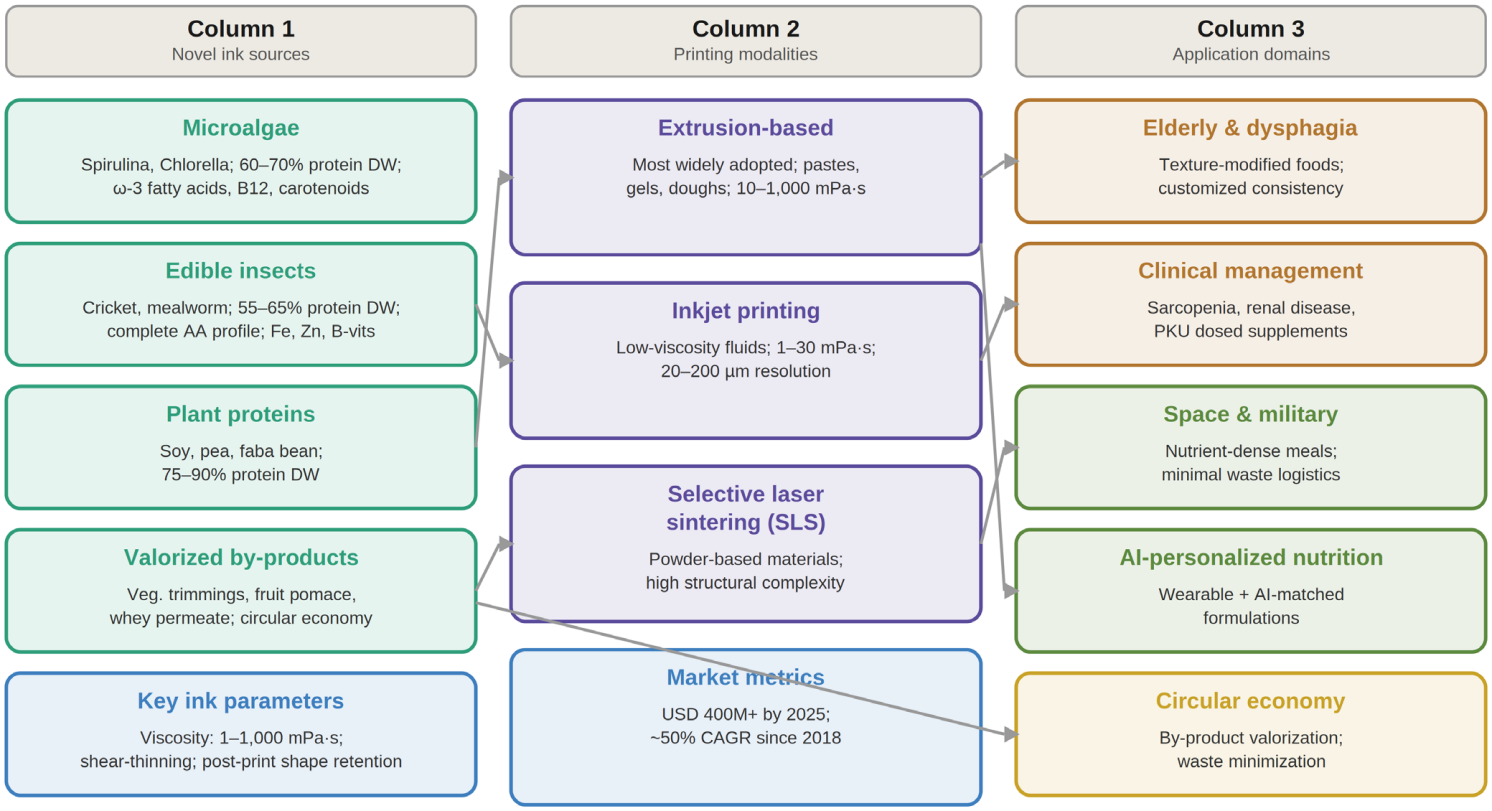
The 3D Food Printing (3DFP) ecosystem. Flow from novel protein ink sources (microalgae, edible insects, plant proteins, valorized by‐products) through digital design and three primary printing modalities (extrusion‐based, inkjet, selective laser sintering) to diverse application domains (elderly and dysphagia nutrition, clinical management, space and military, AI‐personalized nutrition, circular economy). Key rheological parameters and market metrics are indicated. EAA, essential amino acids; PDCAAS, protein digestibility‐corrected amino acid score; SLS, selective laser sintering. *Sources:* Neamah and Tandio (2024); Zhang et al. (2024); Abedini et al. (2024).

The integration of edible insects and microalgae into 3DFP inks represents one of the more innovative intersections of sustainable food science and advanced manufacturing. Insect‐based inks derived from cricket powder ( *Acheta domesticus* ) or mealworm ( *Tenebrio molitor* ) flour are protein‐dense (approximately 55%–65% dry weight) and contain omega‐3 fatty acids, iron, zinc, and B vitamins (Neamah and Tandio 2024; Oonincx and de Boer 2012). Controlled studies incorporating 10%–20% ground insect powder into extruded snacks have reported increases in total essential amino acid content and improved protein digestibility‐corrected amino acid scores; however, the scope of available evidence remains limited to small sample sizes and a narrow range of product types. Microalgae‐based inks contribute protein, natural pigmentation, omega‐3 fatty acids, and antioxidant carotenoids to 3D‐printed products; their incorporation requires careful rheological optimization and flavor masking strategies. Figure 3 illustrates the 3DFP ecosystem from novel inks through printing modalities to end applications.

### 8.3 Personalized Nutrition and Circular Economy Applications

The convergence of 3DFP with wearable health monitoring devices, AI‐driven dietary assessment algorithms, and precision nutrition frameworks envisions individually formulated food products: meals digitally designed to match a user's specific micronutrient requirements, metabolic phenotype, drug‐nutrient interactions, and sensory preferences (Zhang et al. 2024; Abedini et al. 2024). Clinical applications are particularly compelling, including texture‐modified food for patients with dysphagia, precisely dosed protein supplements for sarcopenia management in older adults, and micronutrient‐targeted formulations for patients with renal disease or phenylketonuria (Abedini et al. 2024). From a sustainability perspective, 3DFP can valorize food processing by‐products, including vegetable trimmings, fruit pomace, and whey permeate, as printable bioactive inks, contributing to circular economy principles (Neamah and Tandio 2024). Significant barriers to realizing this vision persist, including the capital cost of food‐grade 3D printing equipment (currently in the range of USD 5000–50,000 per unit), complexity in ensuring microbial safety across diverse printable matrices, limited shelf life of many protein‐rich inks, and regulatory uncertainties around novel insect and algae ingredients in major markets.

## 9 Cross‐Cutting Challenges and Transformative Pathways

### 9.1 Regulatory Harmonization

The global regulatory landscape for next‐generation food technologies is fragmented and rapidly evolving. In the United States, the FDA and USDA share oversight for cultivated meat under a 2019 formal agreement, while EFSA in Europe applies the Novel Foods Regulation (EC 2015/2283) to PF‐derived ingredients and insect proteins. Singapore's SFA was the first authority globally to approve cell‐cultured chicken for commercial sale in December 2020 (Yun et al. 2024). Israel's Ministry of Health approved cultivated beef from Aleph Farms in January 2024, making it the third country to formally permit CM (Yun et al. 2024). These divergent frameworks create barriers to international trade, complicate cross‐border investment, and impede the global diffusion of beneficial technologies. For biofortification specifically, the European Court of Justice's 2018 ruling classifying CRISPR‐edited plants under the full GMO Directive diverges sharply from the US approach of treating many gene‐edited varieties similarly to conventionally bred ones, creating significant market access asymmetries for biofortified crops developed through gene editing (Puchta 2023). Convergence on science‐based, transparent regulatory standards is a priority that transcends the interests of individual jurisdictions.

### 9.2 Consumer Acceptance and Behavior Change

Consumer acceptance is a pivotal determinant of whether next‐generation food technologies achieve the scale required to materially impact global nutrition and sustainability. Neophobia, the psychological aversion to novel or unfamiliar foods, is a well‐documented barrier, particularly for CM and insect‐based products (Kraak and Aschemann‐Witzel 2024). Survey evidence across multiple countries indicates that acceptance increases when products are presented in terms of taste and nutritional equivalence, when labeling communicates safety credentials transparently, and when environmental benefits are communicated clearly and credibly (GFI 2024). However, a critical limitation of the consumer acceptance literature is that surveys of stated intent are weak predictors of actual purchasing behavior; review evidence shows that willingness‐to‐pay premiums for plant‐based alternatives typically fall below the production cost premium of current next‐generation foods, particularly for CM and PF‐derived ingredients (Kraak and Aschemann‐Witzel 2024). Cultural context substantially moderates acceptance; for insect‐based products, acceptance is considerably higher in parts of sub‐Saharan Africa, Southeast Asia, and Latin America where entomophagy is culturally established (Neamah and Tandio 2024; Oonincx and de Boer 2012). Consumer education campaigns and transparent labeling remain evidence‐based strategies, but their translation into sustained purchasing behavior change requires longer‐term evaluation.

### 9.3 Equity, Access, and the LMIC Dimension

A substantive concern in the literature is whether next‐generation food technologies will primarily benefit wealthy consumers in HICs while leaving food‐insecure populations in LMICs further disadvantaged. The capital‐intensive nature of PF and CM currently concentrates research, development, and early commercialization in North America, Europe, and East Asia (Kraak and Aschemann‐Witzel 2024). Conversely, biofortification and edible insect value chains have lower technology barriers and greater immediate applicability in LMIC settings; HarvestPlus has already deployed biofortified varieties on over 221 million hectares in LMICs (Menon and Olney 2024). A more equitable innovation strategy would pursue open‐source strain and crop variety development, South–South technology transfer programs, and deliberate investment in locally relevant approaches including community‐scale fermentation infrastructure (Kraak and Aschemann‐Witzel 2024; Menon and Olney 2024). Intellectual property arrangements that protect corporate returns in HIC markets while foreclosing affordable access in LMICs represent a governance risk that requires proactive policy attention.

### 9.4 Environmental Life Cycle and Energy Considerations

While the environmental credentials of PF and CM are frequently cited as unambiguously superior to conventional animal agriculture, a nuanced life cycle assessment perspective is essential to accurate representation. CM processes are energy‐intensive: maintaining sterile bioreactor environments at appropriate temperatures, with continuous agitation, aeration, and downstream processing, requires substantial electricity inputs. If this energy derives from a fossil fuel‐dependent grid, CM's GHG advantage over conventional beef is substantially reduced or may reverse, as peer‐reviewed LCA data confirm (Risner et al. 2025). Integrating CM and PF production with renewable energy is therefore not an optional enhancement but a prerequisite for the environmental benefits cited in advocacy and scientific literature alike. Future LCA studies should explicitly model a range of energy scenarios and present uncertainty ranges rather than single‐point estimates, apply standardized functional units and system boundaries, and assess end‐of‐life waste streams from bioreactor operation. Table 3 summarizes the major cross‐cutting challenges and proposed transformative pathways.

**TABLE 3:** Key challenges and transformative pathways toward next‐generation food technology adoption.

Challenge	Current Status	Proposed Pathway
Regulatory Uncertainty	Fragmented global standards (EFSA Novel Foods Regulation, US FDA/USDA, Singapore SFA, Israel MoH); CRISPR crop regulations diverge between US and EU	Harmonize international novel food frameworks; publish transparent, evidence‐based approval timelines; clarify CRISPR crop classifications
Consumer Acceptance	Documented neophobia for CM and insect‐based products; willingness‐to‐pay premiums below cost of production; cultural variability in acceptance	Transparent, neutral labeling; independent taste certification; targeted culturally‐adapted education campaigns
Scaling and Cost Parity	PF proteins targeting ~USD 10/kg; CM still at ~USD 63/kg; target<USD 5/kg for price parity	Large‐scale bioreactor engineering investment; serum‐free and plant‐based culture media; AI‐assisted bioprocess optimization (Gu et al. 2025)
Nutritional Completeness	Some plant proteins deficient in essential AAs (e.g., low methionine in pea); micronutrient bioavailability from biofortified crops variable	Complementary protein blending; CRISPR‐aided bioavailability enhancement; nanoencapsulation for improved delivery
Energy Intensity	CM and PF energy‐intensive at industrial scale; fossil‐fuel energy substantially reduces GHG advantage (Risner et al. 2025)	Mandatory integration with renewable energy systems; full‐system LCA with uncertainty ranges as standard reporting
Equity and Access	Technologies concentrated in HICs; LMICs risk exclusion from innovations they most need; IP barriers potential	Open‐source strain development; South–South technology transfer; locally adapted biofortification programs; IP‐access agreements (Menon and Olney 2024)

*Note:* Summary of six major cross‐cutting challenges confronting next‐generation food technologies, with current status assessments and proposed evidence‐based pathways toward resolution.

Abbreviations: AA, amino acid; CM, cultivated meat; HIC, high‐income country; IP, intellectual property; LCA, life cycle assessment; LMIC, low‐and‐middle‐income country; PF, precision fermentation.

## 10 Implications for Global Health and Sustainable Development

The potential public health dividend of mainstreaming next‐generation food technologies is substantial, though it requires calibrated interpretation. At the population level, a dietary transition from conventional animal products toward PF‐derived proteins and plant‐based alternatives aligns with the EAT‐Lancet Commission's planetary health diet, which models that global adoption could prevent approximately 10.9 million premature deaths annually, primarily through reductions in cardiovascular disease, type 2 diabetes, and colorectal cancer attributable to red and processed meat intake (Willett et al. 2019). These projections derive from epidemiological modeling rather than dietary trials, and achievable health benefits would depend critically on the nutritional quality of replacement foods, dietary patterns retained in the overall diet, and implementation context.

The strongest human trial evidence supporting beneficial health outcomes exists for biofortification. Randomized controlled trials across sub‐Saharan Africa and South Asia have demonstrated statistically significant improvements in serum micronutrient status following consumption of iron‐biofortified common beans and vitamin A‐enriched orange‐fleshed sweet potato, translating into measurable reductions in anemia prevalence and improvements in child growth outcomes (Ofori et al. 2022). For mycoprotein, the 2024 RCT by Pavis et al. provides real‐world evidence that regular consumption reduces LDL cholesterol, blood glucose, and c‐peptide concentrations in overweight hypercholesterolemic adults, with effect sizes comparable to major dietary interventions. These represent the strongest controlled human evidence within any of the five pillars.

The global burden of antimicrobial resistance (AMR) is directly linked to the prophylactic and therapeutic use of antibiotics in intensive animal production. Transitioning a material fraction of global protein supply to PF, CM, or plant‐based sources would reduce agricultural antibiotic use, with potential downstream benefits for the efficacy of antimicrobial treatments in human medicine (Soice and Johnston 2021). Quantifying this benefit precisely requires epidemiological modeling of pathogen resistance dynamics that goes beyond current published evidence, and the link between specific dietary transitions and measurable AMR reductions at population scale has not been demonstrated experimentally.

## 11 Future Perspectives and Priority Research Gaps

Convergence across the five technological pillars is occurring in applied research contexts. PF‐derived proteins are being evaluated as precision inks for 3D printing of structured meat analogs, potentially bypassing CM's bioreactor scaffolding challenges. Microalgae cultivated in controlled photobioreactors provide omega‐3‐rich, protein‐dense inks for personalized functional food products. Machine learning and AI are being applied to bioprocess optimization, dietary needs assessment, and sensory profile matching (Zhang et al. 2024; Abedini et al. 2024). These convergence trends are promising but largely at early demonstration phase; translation to commercial scale requires validation at each step.

Priority research gaps identified in this review are: (i) comparative, full‐system LCA studies incorporating renewable energy scenarios, standardized functional units, and explicit uncertainty ranges for PF and CM; (ii) long‐term (minimum 12‐month) dietary intervention trials evaluating the nutritional adequacy and health outcomes of diets substantially derived from next‐generation proteins across diverse demographic and geographic populations; (iii) participatory, co‐design research with LMIC communities to ensure that biofortification programs and novel food technologies address locally relevant nutritional priorities, are culturally acceptable, and are economically accessible; (iv) techno‐economic analyses of 3DFP deployment in clinical nutrition, institutional catering, and emergency relief settings; (v) independent citation accuracy and bibliometric audits for rapidly evolving technology fields, where AI‐assisted literature synthesis has been documented to generate non‐traceable or fabricated references, raising serious concerns for the integrity of the scientific record; and (vi) robust regulatory science that provides internationally harmonized, evidence‐based approval pathways proportionate to demonstrated risk.

Interdisciplinary collaboration spanning food science, microbiology, synthetic biology, tissue engineering, nutrition epidemiology, behavioral economics, regulatory science, environmental sustainability, and agronomy is a prerequisite for generating the rigorous, reproducible evidence base that high‐stakes food system decisions require.

## 12 Conclusion

This review has critically examined five next‐generation food technologies—precision fermentation, cellular agriculture, plant‐based and mycoprotein innovations, biofortification, and 3D food printing—as components of a potential transformative response to the global food‐nutrition‐sustainability challenge. Each pillar offers distinct and scientifically supported contributions: PF enables ecologically efficient production of animal‐identical proteins under defined conditions; CM decouples meat from animal slaughter and associated zoonotic risk; plant‐based and mycoprotein systems provide scalable, lower‐impact protein sources backed by growing clinical evidence (Pavis et al. 2024); biofortification delivers micronutrients at population scale with the strongest randomized trial evidence base of any pillar (Ofori et al. 2022); and 3DFP enables personalized, waste‐minimizing nutrition with compelling potential for clinical applications.

Critically, however, each technology carries documented scientific, technical, regulatory, and socioeconomic uncertainties that are frequently underemphasized in technology‐optimistic projections. These uncertainties encompass: the dependence of LCA advantages on energy decarbonization that has not yet occurred at the required scale (Risner et al. 2025); the complete absence of long‐term human dietary trials for diets substantially derived from PF‐ or CM‐sourced foods; the unresolved equity implications of capital‐intensive technologies concentrated in wealthy economies; the regulatory barriers facing gene‐edited biofortified crops in the European Union; the sensitivity of market growth projections to investment climates, regulatory decisions, and consumer adoption trajectories that are inherently difficult to forecast; and the documented risk of unverified citation claims in rapidly evolving fields where AI‐assisted synthesis has been used without adequate bibliographic verification.

No single technology will resolve global food insecurity. The most credible pathway toward a nutritionally adequate and ecologically viable food future is a scientifically rigorous, equitably governed, and evidence‐anchored portfolio approach—one that deploys each technology where it offers the greatest comparative advantage, ensures that the benefits are accessible across income levels, and maintains public trust through transparency, independent safety evaluation, and honest communication of both potential and limitations. The next‐generation nutritional frontiers are not a distant prospect; they are a present scientific and governance imperative.

## Author Contributions

**Adefila Adebimpe Moyosore:** conceptualization, writing – original draft, investigation, supervision, writing – review and editing. **Kareem Sarafadeen Olateju:** writing – original draft, writing – review and editing. **Zakari Adeiza David:** conceptualization, software, investigation, writing – original draft, writing – review and editing, project administration, resources, supervision, methodology, validation, data curation, formal analysis. **Audu Godwin Amoka:** conceptualization, methodology, writing – review and editing, formal analysis. **Mustapha Omenesa Idris:** conceptualization, writing – review and editing, formal analysis, software.

## Ethics Statement

The authors have nothing to report.

## Consent

The authors have nothing to report.

## Conflicts of Interest

The authors declare no conflicts of interest.

## Data Availability

Data sharing is not applicable to this article as no new data were created or analyzed. This is a review article and all referenced data are available in the original publications cited herein.
